# Exploratory analysis of depressive symptom trajectories before and after hip or knee arthroplasty in geriatric patients

**DOI:** 10.1007/s00402-026-06268-6

**Published:** 2026-03-28

**Authors:** Julia Schiegl, Philip Bammert, Günther Maderbacher, Jan Reinhard, Stefano Pagano, Katrin Michalk, Joachim Grifka, Tobias Kappenschneider

**Affiliations:** 1 Department of Orthopaedics, University Hospital Regensburg, Regensburg, Germany; 2 Department of Health Economics, Technical University of Munich, Munich, Germany; 3 Orthopaedics and Ergonomics Research Centre, East Bavarian Technical University (OTH), Regensburg, Germany

**Keywords:** Orthogeriatric, Geriatric Depression Scale (GDS-15), Psychological well-being, Pain–depression interaction, Postoperative recovery

## Abstract

**Background:**

Depressive symptoms are prevalent among patients with osteoarthritis (OA), particularly in geriatric patients. Pain and mood are closely interconnected, with chronic joint pain contributing significantly to psychological distress. Total hip arthroplasty (THA) and total knee arthroplasty (TKA) are established procedures that improve pain and physical function and may also influence depressive symptoms. The objective of this study was to evaluate changes in depressive symptoms following THA and TKA in geriatric patients.

**Methods:**

In this prospective pilot study, we analysed data from 143 participants enrolled in the ongoing Special Orthopaedic Geriatrics (SOG) trial, funded by the German Federal Joint Committee (G-BA). Depressive symptoms were assessed using the Geriatric Depression Scale (GDS) preoperatively and at 3 days, 7 days, 4 weeks, and 3 months after surgery. Depressive symptoms were analysed in two predefined groups: the total sample (GDS 1–15) and patients with elevated baseline symptoms (GDS 6–15). Statistical analysis included the Friedman test for repeated measures, followed by post-hoc testing.

**Results:**

In the overall cohort, median GDS scores decreased from 3 at baseline to 2 at the 3-month follow-up (*p* < 0.001). In the subgroup with elevated baseline symptoms (GDS 6–15), median scores declined from 8.5 at baseline to 4 at 3 months (*p* < 0.001). Improvements were observed after both THA and TKA, with changes appearing more pronounced in THA.

**Conclusion:**

THA and TKA were associated with modest improvements in depressive symptoms across the full range of baseline GDS scores. While patients with elevated baseline symptoms showed larger absolute changes, improvements were also observed in those without abnormal baseline scores. Improvements in depressive symptoms were most pronounced between the preoperative and early postoperative assessments, whereas only minor additional changes were observed during later postoperative follow-up. These findings should be interpreted as exploratory and require confirmation in larger controlled studies.

**Trial registration:**

This study is part of the Special Orthopaedic Geriatrics (SOG) trial, German Clinical Trials Register DRKS00024102. Registered on 19 January 2021.

**Supplementary Information:**

The online version contains supplementary material available at 10.1007/s00402-026-06268-6.

## Background

The number of geriatric patients with osteoarthritis (OA) is expected to rise significantly in the coming decades [5]. Progressive joint degeneration in OA often leads to a loss of function and ultimately culminates in chronic pain [16]. When conservative treatment options have been exhausted, total hip arthroplasty (THA) and total knee arthroplasty (TKA) represent the gold standard for managing advanced OA [20]. According to projections, the number of primary hip and knee replacements in the United States is expected to increase by 176% and 139% respectively by 2040, and by 659% and 469% respectively by 2060 [35]. In Germany, the number of primary hip and knee arthroplasties is likewise expected to rise substantially, with projected increases of approximately 45% for hip replacements and 55% for knee replacements by 2040, driven by demographic ageing and increased life expectancy. This global and national expansion is closely linked to demographic trends, with the number of geriatric patients rising substantially [40].

It is important to emphasize that geriatric patients differ significantly from the average orthopaedic population. They often present with multiple age-related challenges, including multimorbidity, malnutrition, polypharmacy, osteoporosis, immobility, dementia, and other geriatric syndromes [3, 26]. Both aging- and disease-related processes contribute to an increased vulnerability to depressive symptoms, which frequently emerge as a response to compromised physical, mental, or social well-being [2]. Evidence shows that preoperative depressive symptoms are associated with poorer clinical outcomes one year after THA and TKA [10]. Persistent depressive symptoms can impair social functioning, compromise self-care, and either resemble cognitive decline (pseudodementia) or worsen pre-existing dementia [18]. Therefore, early recognition of depressive symptoms in elderly patients is crucial, enabling timely and appropriate intervention by the treatment team [31].

A widely used tool to assess depressive symptoms in geriatric patients is the 15-item Geriatric Depression Scale (GDS-15) [43]. Numerous risk factors for depression in this population have been identified, including advanced age, multimorbidity, obesity, polypharmacy, and low levels of physical activity [23]. Osteoarthritis - through its restriction of mobility and associated decline in quality of life - has been linked to both depressive symptoms and increased cardiovascular mortality [27]. While THA and TKA have been shown to improve quality of life, mobility, and depressive symptoms in average orthopaedic patients [10], geriatric patients remain markedly underrepresented in existing studies. Many previous investigations have focused on younger or mixed-age cohorts, often excluding individuals aged ≥ 75 years or those with multimorbidity. Consequently, evidence specifically addressing depressive symptoms in elderly arthroplasty patients is scarce. This underrepresentation underscores a critical gap in the literature and highlights the need for studies focusing exclusively on elderly patients undergoing THA or TKA [21].

### Aim of the study

In this prospective observational study, we aimed to explore whether THA and TKA are associated with changes in depressive symptoms among geriatric patients at several defined postoperative time points (3 days, 7 days, 4 weeks, and 3 months). We hypothesized that both procedures would be associated with measurable postoperative decreases in depressive symptoms as assessed by the GDS-15. Furthermore, we sought to compare depressive symptom scores across these time points to describe the trajectory of postoperative changes. To our knowledge, no previous study has examined this association specifically in a geriatric population of comparable age and multimorbidity.

## Methods

### Study design

This investigation was conducted as a prospective observational pilot substudy embedded within the ongoing Special Orthopaedic Geriatrics (SOG) trial, registered in the German Clinical Trials Register (DRKS00024102). The study was approved by the local ethics committee (file number 20–1680−101). The overarching SOG trial evaluates a multimodal orthogeriatric care model described in detail elsewhere [13–15]. The designation as a pilot substudy reflects the exploratory nature of the analysis, the absence of an a priori sample size calculation, and the limited subgroup sizes available for inferential testing.

Participants were consecutively recruited from the orthopaedic outpatient clinic of a tertiary care centre with high annual arthroplasty volume, where they were screened for eligibility by the study team immediately prior to inclusion. All patients fulfilling the clinical indication for THA or TKA and meeting the eligibility criteria during the recruitment period were approached. The exact numbers of screened, eligible, excluded, and included patients are reported in the Results section.

The study adhered to the principles of the Declaration of Helsinki. All procedures involving human participants were conducted in accordance with the Declaration of Helsinki [42]. All participants provided written informed consent, and the study protocol received approval from the institutional ethics committee.

### Study population

Eligible patients were scheduled for elective THA or TKA due to primary OA of the hip or knee and were aged ≥ 80 years regardless of multimorbidity, or 70–79 years in the presence of multimorbidity. Patients aged 70–79 years without multimorbidity were not eligible. Exclusion criteria comprised age < 70 years, acute infections, previous bone surgery of the affected joint, tumor in the operative region, or extensive need for nursing care.

Furthermore, a Mini-Mental State Examination (MMSE) score below 16 constituted an exclusion criterion, as adequate cognitive ability was necessary for a valid GDS assessment [39]. The MMSE was administered preoperatively by trained study nurses and interpreted using age-adjusted normative values, ensuring culturally and linguistically appropriate score interpretation.

### Data collection

Data were collected at five predefined time points: preoperatively (T0), postoperative day 3 (T1), postoperative day 7 (T2), 4 weeks (T3), and 3 months postoperatively (T4). These intervals were selected to capture both early postoperative responses and improvements occurring during the subacute rehabilitation period typical for geriatric arthroplasty patients.

### Surgical procedures and postoperative management

All surgical procedures were performed in a tertiary care centre with high annual arthroplasty volume, using standardized perioperative protocols.

Patients were mobilized under full weight-bearing from the first postoperative day onward and received daily physiotherapy for four weeks, delivered either in the acute hospital, a rehabilitation clinic, or in outpatient settings.

TKA procedures were performed using a medial parapatellar approach with cemented PFC Sigma components (DePuy, Warsaw, IN, USA), without patellar resurfacing. THA procedures were performed using a minimally invasive anterolateral approach in the lateral decubitus position, employing press-fit acetabular components and cementless Corail femoral stems (DePuy Synthes, Warsaw, IN, USA).

### Functional and comorbidity assessment

To comprehensively characterize the health status, frailty, and functional baseline of the study population, a multimodal geriatric assessment was administered preoperatively, as recommended for orthogeriatric research.

Overall comorbidity burden was assessed using the Charlson Comorbidity Index (CCI) derived from documented comorbidities using an ICD-10-based coding approach (The CCI is an index; it is not a language-dependent questionnaire); higher values indicate increased mortality risk and the non–age-adjusted CCI theoretically ranges from 0 to 37 points [6, 32, 43].

Functional independence was evaluated using the Lawton–Brody Instrumental Activities of Daily Living (IADL) Scale, ranging from 0 (complete dependence) to 8 (complete independence) [19]. A German translation (word-for-word) was used for administration.

Frailty status was assessed using the Physical Frailty Phenotype (PFP) according to Fried et al., evaluating unintentional weight loss, exhaustion, grip strength, gait speed, and physical activity. Scores range from 0 to 5, with scores ≥ 3 indicating frailty [34, 45].

Lower extremity function was measured using the Short Physical Performance Battery (SPPB), which assesses balance, gait speed, and chair-stand performance (range 0–12; higher scores reflect better physical function) [11]. A German instruction sheet (word-for-word) was used for administration.

Body mass index (BMI) was measured by healthcare professionals during the preoperative assessment [28].

The Nutritional Risk Screening (NRS) was used to assess the risk of malnutrition based on nutritional status (weight loss, BMI, and dietary intake) and disease severity. The screening consists of two components (nutritional impairment and severity of illness), each scored between 0 and 3, with an additional point added for age ≥ 70 years. Total scores range from 0 to 7, with scores ≥ 3 indicating a clinically relevant risk of malnutrition. The NRS-2002 was administered using the German-language version described by Schütz et al. [34]. The original NRS-2002 development paper by Kondrup et al. was cited as the primary source for the instrument [17].

The Barthel Index was used to quantify performance in basic activities of daily living, including feeding, bathing, grooming, dressing, continence, toileting, transfers, and mobility. Scores range from 0 (complete dependence) to 100 (complete independence), with higher values indicating better functional status. A validated German version of the Barthel Index was used [12].

### Assessment of depressive symptoms

Depressive symptoms were assessed using the validated German version of the GDS-15, a screening instrument designed specifically for elderly populations and suitable for individuals with mild cognitive impairment [9, 36, 43].

The questionnaire items are presented in Table 1. Each item is answered with “yes” or “no.” Items 1, 5, 7, 11, and 13 are scored with one point for a “no” answer; all others score one point for “yes.” Total scores range from 0 to 15, with scores ≥ 6 indicating depressive symptoms and scores ≥ 11 indicating clinically significant depressive symptomatology [9, 43]. All questionnaires, including the GDS-15, were administered by trained study staff (geriatric nurses or research assistants) under the supervision of a geriatrician and investigator. Self-report was offered when appropriate.

**Table 1:** Items of the 15-item geriatric depression scale (GDS-15)

Item No.	Question
1	Are you basically satisfied with your life?
2	Have you dropped many of your activities and interests?
3	Do you feel that your life is empty?
4	Do you often get bored?
5	Are you in good spirits most of the time?
6	Are you afraid that something bad is going to happen to you?
7	Do you feel happy most of the time?
8	Do you often feel helpless?
9	Do you prefer to stay at home, rather than going out and doing things?
10	Do you feel that you have more problems with memory than most?
11	Do you think it is wonderful to be alive now?
12	Do you feel worthless the way you are now?
13	Do you feel full of energy?
14	Do you feel that your situation is hopeless?
15	Do you think that most people are better off than you are?

Based on established literature and commonly used cut-off values, three GDS-based subgroups were defined a priori:

- the total cohort (GDS 1–15),
- patients with abnormal depressive symptoms (GDS 6–15),
- patients with clinically significant symptoms (GDS 11–15).

The GDS 11–15 subgroup was defined descriptively but was not included in inferential statistical analyses due to the small sample size.

### Statistical analysis

Descriptive statistics were used to summarize demographic and clinical characteristics. Because GDS scores are ordinal and non-normally distributed, the Friedman test was selected to examine changes across time points. Medians and interquartile ranges are reported.

When the Friedman test indicated a significant overall change, post-hoc pairwise comparisons were performed using Wilcoxon signed-rank tests with Bonferroni correction. Subgroup analyses for THA and TKA were conducted using the same statistical approach. Subgroups with fewer than 10 participants were considered too small for reliable post-hoc inferential testing. Effect sizes for Wilcoxon signed-rank tests were interpreted as small (*r* = 0.10–<0.30), moderate (*r* = 0.30–<0.50), and large (*r* ≥ 0.50) [8].

All analyses were performed using R (R Foundation for Statistical Computing, Vienna, Austria), version 4.2.1, and the rstatix package. Statistical significance was defined as *p* < 0.05.

## Results

### Baseline characteristics

**Fig. 1:**
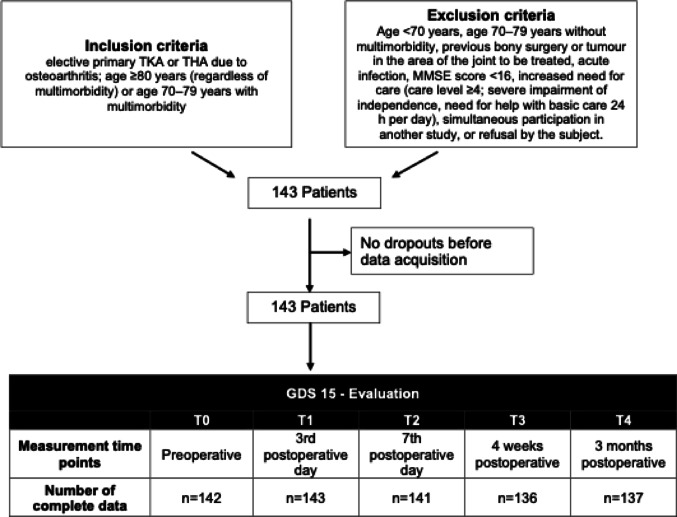
Flowchart of study group enrolment.* Care level ≥ 4 according to the German statutory long-term care insurance classification (Social Security Code XI*,* Care Strengthening Act II); severe impairment of independence*,* need for help with basic care 24 h per day*

A total of 143 participants were included in the analysis, comprising 84 individuals who underwent THA and 59 who underwent TKA. There were no complete dropouts. In one case, the GDS could not be administered preoperatively, and in two cases it was not completed on postoperative day 7. Loss to follow-up occurred in six participants at the four-week assessment and in five participants at the three-month follow-up (Fig. 1).

Women represented 65.03% of the study population (Table 2). The mean age was 78.57 ± 4.66 years, and the mean BMI was 28.91 ± 4.91 kg/m². Participants were taking an average of 7.60 ± 3.60 medications and presented with 7.70 ± 3.02 comorbidities. The mean CCI was 5.33 ± 1.87. The NRS score averaged 1.27 ± 0.76, and functional performance was reflected by a Barthel Index of 92.87 ± 11.44, an IADL score of 6.71 ± 1.59, and a SPPB of 6.98 ± 2.74. Frailty, as assessed by the PFP, averaged 2.18 ± 1.28, and cognitive status measured by the MMSE averaged 26.80 ± 2.65.

**Table 2:** Baseline characteristics of the 143 patients

	All	Hip	Knee
Characteristics	*n* = 143	84	59
Female n (%)	93 (65.03%)	54 (64,29%)	39 (66,1%)
Age y, mean ± SD	78.57 ± 4.66	78.21 ± 4.51	79.08 ± 4.85
BMI kg/m² mean ± SD	28.91 ± 4.91	28.59 ± 5.40	29.35 ± 4.10
Medication n mean ± SD	7.60 ± 3.60	7.51 ± 3.65	7.73 ± 3.56
Comorbidities n, mean ± SD	7.70 ± 3.02	7.39 ± 3.02	8.13 ± 2.99
CCI mean ± SD	5.33 ± 1.87	5.35 ± 2.03	5.31 ± 1.64
NRS Score (0–7) mean ± SD	1.27 ± 0.76	1.35 ± 0.81	1.15 ± 0.66
Barthel Index (0–100), mean ± SD	92.87 ± 11.44	92.26 ± 11.81	93.73 ± 10.03
IADL score (0–8) mean ± SD	6.71 ± 1.59	6.54 ± 1.66	6.97 ± 1.45
PFP score (0–5), mean ± SD	2.18 ± 1.28	2.49 ± 1.20	1.74 ± 1.26
SPPB score (0–12) mean ± SD	6.98 ± 2.74	6.74 ± 2.77	7.33 ± 2.66
MMSE score (0–30) mean ± SD	26.80 ± 2.65	26.93 ± 2.76	26.61 ± 2.48

*SD*,* Standard deviation; BMI*,* Body mass index; CCI*,* Charlson comorbidity index; NRS*,* Nutritional risk screening; IADL*,* Instrumental activities of daily living; PFP*,* Physical frailty phenotype (Fried); MMSE*,* Mini-mental state examination; SPPB*,* Short physical performance battery*

A significant improvement in depressive symptoms between the preoperative and postoperative assessments was observed across the cohort (Friedman test, Table 3). Median GDS was reported separately for subgroups with scores 6–11 and 11–15. However, no post-hoc analyses were conducted for these subgroups due to insufficient sample sizes. The GDS 11–15 subgroup was reported descriptively because of the very small sample size; the Friedman test p-value shown in Table 3 is provided for completeness but was not used for inferential interpretation. The median GDS score (range 1–15) decreased from 3 at T0 to 2 at T4 (*p* < 0.001). In individuals with clinically relevant depressive symptoms (GDS 6–15), the median score declined from 8.5 at T0 to 4 at T4, representing a reduction of more than 50% (*p* < 0.001). The postoperative trajectories for THA and TKA are presented separately in Table 3 to illustrate procedure-specific patterns of change.

**Table 3:** Median Geriatric Depression Scale (GDS-15) scores and interquartile ranges at five measurement time points (T0–T4)

A) Total sample GDS Score						
	T0 *Md(IQR)*	T1 *Md(IQR)*	T2 *Md(IQR)*	T3 *Md(IQR)*	T4 *Md(IQR)*	*p-value*
GDS Score 1-15	3(1-4)	2(1-4)	2(1-3)	2(1-3)	2(1-3)	**<0.001**
GDS Score 6-15	8.5(7-10)	7(5-8)	6(3-9)	5(2,5-9)	4(3-9)	**<0.001**
GDS Score 11-15	12(12-12)	8(8-8)	9(8-9)	8(4,75-10,25)	9(8-10)	0.079

B) GDS hip arthoplasty group						
	T0 *Md(IQR)*	T1 *Md(IQR)*	T2 *Md(IQR)*	T3 *Md(IQR)*	T4 *Md(IQR)*	*p-value*
GDS Score 1-15	3(1-4.25)	2(1-4)	2(1-3)	2(1-3)	2(1-3)	**<0.001**
GDS Score 6-15	8(7-9.5)	7(5.5-8)	7(3-9)	5.5(3-10)	4(3-9)	0.102

B) GDS Knee arthoplasty group						
	T0 *Md(IQR)*	T1 *Md(IQR)*	T2 *Md(IQR)*	T3 *Md(IQR)*	T4 *Md(IQR)*	*p-value*
GDS Score 1-15	2(1-4)	2(1-4)	2(1-3)	2(1-3)	2(1-3)	**<0.001**
GDS Score 6-15	9(7-11)	7(5-8)	5(4-7)	5(2-6)	4(3-7)	**0.001**

T0 = preoperative, T1 = 3rd postoperative day, T2 = 7th postoperative day, T3 = 4 weeks postoperative, and T4 = 3 months postoperative. Md = Median; IQR = Interquartile Range; p = p-value of the Friedman Test. A) Total sample, B) Hip arthroplasty group, C) Knee arthroplasty group

### Post-hoc analyses in the overall cohort

The results of the Wilcoxon signed-rank post-hoc tests for all pairwise time-point comparisons are shown in Table 4. Significant decreases in GDS scores were observed between T0 and T2 (*p* < 0.001), T0 and T3 (*p* < 0.001), and T0 and T4 (*p* < 0.001). After Bonferroni correction, the comparison between T0 and T1 was not statistically significant (*p* = 0.038). No significant differences were identified between any of the postoperative time points (all *p* > 0.05).

Effect sizes were moderate to large for comparisons between preoperative and postoperative assessments, whereas effect sizes between postoperative time points were small (Table 4). Effect sizes were small for T0 vs. T1 (*r* = 0.241) and moderate for T0 vs. T2–T4 (*r* = 0.387–0.493), while postoperative comparisons showed small to negligible effects (*r* = 0.027–0.297).

**Table 4:** Post-hoc comparisons of Geriatric Depression Scale (GDS-15) scores across measurement time points (T0–T4).

A) Total sample GDS Score					
contrast	T0 vs T1	T0 vs T2	T0 vs T3	T0 vs T4	T1 vs T2
p*	**0.038**	**<0.001**	**<0.001**	**<0.001**	0.070
r	0.241	0.387	0.467	0.493	0.212

contrast	T1 vs T3	T1 vs T4	T2 vs T3	T2 vs T4	T3 vs T4
p*	**0.006**	**0.026**	1	0.687	1
r	0.297	0.275	0.094	0.162	0.027

B) GDS hip arthoplasty group					
contrast	T0 vs T1	T0 vs T2	T0 vs T3	T0 vs T4	T1 vs T2
p*	**0.011**	**<0.001**	**<0.001**	**<0.001**	0.026
r	0.282	0.458	0.422	**0.507**	0.221

contrast	T1 vs T3	T1 vs T4	T2 vs T3	T2 vs T4	T3 vs T4
p*	**0.023**	**0.007**	0.970	0.241	0.339
r	0.255	0.319	0.002	0.143	0.125

C) GDS Knee arthoplasty group					
contrast	T0 vs T1	T0 vs T2	T0 vs T3	T0 vs T4	T1 vs T2
p*	1	0.306	**0.001**	**0.010**	1
r	0.188	0.288	**0.531**	0.470	0.211

contrast	T1 vs T3	T1 vs T4	T2 vs T3	T2 vs T4	T3 vs T4
p*	0.074	1	1	1	1
r	0.364	0.216	0.224	0.191	0.107

T0 = preoperative, T1 = 3rd postoperative day, T2 = 7th postoperative day, T3 = 4 weeks postoperative, T4 = 3 months postoperative. Bonferroni correction applied for 10 comparisons; p = p-value from the pairwise Wilcoxon signed-rank test; r = effect size. A) Total sample, B) Hip arthroplasty group, C) Knee arthroplasty group

### Subgroup analyses by baseline depressive symptom severity

Table 5 presents the trajectory analyses stratified by baseline GDS severity. In the subgroup with abnormal depressive symptoms (GDS 6–15), significant improvements were observed between T0 and T1 (*p* = 0.010), T0 and T2 (*p* = 0.014), T0 and T3 (*p* = 0.005), and T0 and T4 (*p* = 0.018). No significant differences were observed among postoperative time-point comparisons (all *p* > 0.05). In the GDS 6–15 subgroup, all preoperative-to-postoperative contrasts showed large effects (T0 vs. T1–T4: *r* = 0.677–0.734), whereas postoperative contrasts were small to negligible (*r* = 0.022–0.309).

**Table 5:** Post-hoc comparisons of Geriatric Depression Scale (GDS-15) scores across measurement time points (T0–T4).

contrast	T0 vs T1	T0 vs T2	T0 vs T3	T0 vs T4	T1 vs T2
p*	**0.010**	**0.014**	**0.005**	**0.018**	1
r	**0.686**	**0.697**	**0.734**	**0.677**	0.204

contrast	T1 vs T3	T1 vs T4	T2vs T3	T2 vs T4	T3 vs T4
p*	1	1	1	1	1
r	0.309	0.234	0.087	0.115	0.022

T0 = preoperative, T1 = 3rd postoperative day, T2 = 7th postoperative day, T3 = 4 weeks postoperative, T4 = 3 months postoperative. Bonferroni correction applied for 10 comparisons; p = p-value from the pairwise Wilcoxon signed-rank test; r = effect size. A) Total sample GDS 6-15

### Stratified analyses by surgery type

In the THA subgroup, significant reductions in depressive symptoms were found between T0 and all postoperative assessments: T1 (*p* = 0.011), T2 (*p* < 0.001), T3 (*p* < 0.001), and T4 (*p* < 0.001). In contrast, the TKA subgroup showed significant improvements only between T0 and T3 (*p* = 0.001) and between T0 and T4 (*p* = 0.010). No other time-point comparisons reached statistical significance (all *p* > 0.05). These trajectories are presented in supplementary figures S1 and S2.

## Discussion

The most important finding of this pilot study is that elderly patients with elevated baseline depressive symptoms (GDS 6–15) may experience postoperative improvements following THA or TKA, with the largest changes occurring between the preoperative and early postoperative assessments.

Pain and depressive symptoms are known to influence each other bidirectionally, which may help explain why patients with OA exhibit higher rates of depressive symptoms than the general population [22]. While depressive symptoms have frequently been viewed as predictors of adverse surgical outcomes, our prospective observational pilot study examined the reverse perspective - evaluating the effect of primary THA and TKA on depressive symptoms in 143 elderly patients. We observed statistically significant improvements in GDS scores beginning within the first postoperative week, with effects persisting through the 3-month follow-up. Overall, improvements were most pronounced between the preoperative and early postoperative assessments, whereas changes between postoperative follow-up time points were small.

Effect sizes indicated small to moderate changes in the overall cohort, while several comparisons within the GDS 6–15 subgroup showed moderate to large effects, suggesting a stronger postoperative response in patients with elevated baseline symptoms. These findings align with earlier studies reporting reductions in depressive symptoms after arthroplasty in mixed-age populations [4, 7, 29].

Patients with preoperative depressive symptom levels (GDS 6–15) appeared to benefit particularly from arthroplasty. The subgroup with severe baseline depressive symptoms (GDS 11–15) was very small and was therefore not included in inferential analyses; descriptive observations are addressed in the Results section. Severely depressed patients are often underrepresented in elective arthroplasty cohorts, as severe psychiatric comorbidities are associated with increased perioperative risk and thus may render individuals less suitable candidates for elective joint replacement [37].

Despite challenges in screening depressive symptoms in geriatric patients, validated tools such as the GDS-15 allow for reliable detection [43]. A validated German version of the GDS-15 was used in our study [9, 43], and the applied cut-offs (≥ 6 and ≥ 11) reflect commonly used thresholds described in the literature. The relevance of screening is underscored by studies showing associations between psychiatric comorbidities and adverse outcomes, including increased postoperative complications, readmission, and higher healthcare costs [30, 41, 44].

Osteoarthritis is the most common chronic joint disease globally and a major contributor to psychological distress due to persistent pain [35]. Pain may trigger depressive symptoms and depression may amplify pain perception [25]. This dynamic interplay highlights the need to consider both physical and psychological aspects in surgical decision-making.

Differences between THA and TKA outcomes in our study may reflect known disparities in postoperative pain, functional recovery, and patient satisfaction. THA patients typically experience faster gait recovery, less postoperative pain, and higher satisfaction, which may positively influence postoperative psychological well-being [33].

Barbosa et al. reported improvements in pain, function, and depressive symptoms after TKA across age groups [4], while Pérez-Prieto et al. found reductions in depressive symptoms following both THA and TKA [29]. Our study extends these findings by focusing on a uniquely geriatric population with a mean age of 78.6 years - substantially older and more multimorbid than in most previous arthroplasty research [1].

Given the high prevalence of depressive symptoms among adults ≥ 70 years and the association with increased all-cause and cardiovascular mortality [24], both preventive and therapeutic strategies are essential. Our findings suggest that depressive symptoms in elderly individuals may be more modifiable than previously assumed, although these results should be interpreted within the context of a pilot study. More research is required to explore mechanisms driving postoperative mood changes, including pain relief, mobility restoration, psychosocial factors, and inflammation. The recent study by Tamate et al. [38] reinforces the importance of mental health in early postoperative outcomes.

Despite growing awareness, no international consensus exists on the definition and assessment of depressive symptoms in geriatric patients. The subgroup definitions were based on literature-derived cut-offs and were not self-constructed.

### Limitations

This study has several limitations. As a secondary analysis within an ongoing trial, it was not part of the predefined aims, and no a priori sample size calculation was performed, which supports its classification as a pilot study. A control group was not included. All patients had exhausted conservative therapies - a standard clinical pathway in geriatric OA care and therefore not a limitation in terms of generalizability - but this precludes assessing the isolated effect of surgery without the influence of prior multimodal treatments.

Not all instruments used in this substudy are available as psychometrically validated German questionnaire versions. The Lawton–Brody IADL and the SPPB were administered using German word-for-word translations and standardized instruction sheets to ensure uniform application. The NRS was applied using the German-language version described by Schütz et al., while the primary instrument source is the original NRS-2002 publication by Kondrup et al. In contrast, the CCI was derived from ICD-10-coded comorbidities and therefore represents a coding-based index rather than a language-dependent questionnaire. These differences in instrument versions and operationalization should be considered when comparing absolute values across studies. In addition, some assessment instruments (frailty and functional measures) lack formal validation in German, and German normative data for the MMSE are unavailable, which may limit comparability. Nevertheless, all applied assessments are routinely used in clinical care and research and are endorsed within the German S3 guideline on Comprehensive Geriatric Assessment (CGA) and by the Medical Service of the Health Insurance Funds [7].

Physical performance was not systematically analyzed. Postoperative physiotherapy, which was part of routine care, may have contributed indirectly to psychological changes; however, its specific impact cannot be quantified within the present design.

Small sample sizes in the GDS 6–15 subgroups for both THA and TKA, and particularly in the GDS 11–15 subgroup, limit statistical power and prevent meaningful subgroup-specific post-hoc analyses. Only short-term postoperative outcomes were assessed; no one-year follow-up data are available, which restricts conclusions about long-term trajectories.

### Strengths

A major strength is the prospective design in a geriatric cohort with a mean age of nearly 79 years and relatively high multimorbidity. Orthogeriatric co-management enabled the use of comprehensive geriatric assessment tools, and data analysis was conducted independently, ensuring objectivity.

## Conclusion

In this pilot study, THA and TKA were associated with short-term improvements in emotional well-being among elderly patients with hip or knee OA. Elderly patients with elevated baseline depressive symptoms (GDS 6–15) appeared to benefit, supported by moderate to large effect sizes for preoperative-to-postoperative comparisons. Future studies should incorporate mechanistic analyses (e.g., pain trajectories, mobility recovery, psychosocial mediators, inflammatory markers) and long-term follow-up to better understand pathways influencing postoperative depressive symptoms.

## Supplementary Information

Below is the link to the electronic supplementary material.

Supplementary Material 1

## Data Availability

On request, data is available at the authors’ institution.
