# Supplementary material for: Exploratory analysis of depressive symptom trajectories before and after hip or knee arthroplasty in geriatric patients
Source: Arch Orthop Trauma Surg. 2026 Mar 28;146(1):125. doi: 10.1007/s00402-026-06268-6 (PMC13032937; doi:10.1007/s00402-026-06268-6)
Supplement: Supplementary file 1 — Supplementary Material 1 [file 402_2026_6268_MOESM1_ESM.docx]

******

***Supplementary Figure S1:*** *GDS Score 1-15 Boxplot of the ‑ total study group, knee arthroplasty and hip arthroplasty ‑ subgroup at five measurement time points (T0–T4). T0 = preoperative, T1 = 3rd postoperative day, T2 = 7th postoperative day, T3 = 4 weeks postoperative, and T4 = 3 months postoperative. Grey dots indicate individual observations; Black dots indicate outliers*

***Supplementary Figure S2****: GDS Score 6-15 Boxplot of the ‑ total study group, knee arthroplasty and hip arthroplasty ‑ subgroup at five measurement time points (T0–T4). T0 = preoperative, T1 = 3rd postoperative day, T2 = 7th postoperative day, T3 = 4 weeks postoperative, and T4 = 3 months postoperative. Grey dots indicate individual observations; Black dots indicate outliers.*
